# Excellent local control with IOERT and postoperative EBRT in high grade extremity sarcoma: results from a subgroup analysis of a prospective trial

**DOI:** 10.1186/1471-2407-14-350

**Published:** 2014-05-20

**Authors:** Falk Roeder, Burkhard Lehner, Thomas Schmitt, Bernd Kasper, Gerlinde Egerer, Oliver Sedlaczek, Carsten Grüllich, Gunhild Mechtersheimer, Patrick Wuchter, Frank W Hensley, Peter E Huber, Juergen Debus, Marc Bischof

**Affiliations:** 1 Clinical Cooperation Unit Radiation Oncology, German Cancer Research Center (DKFZ), Heidelberg, Germany; 2 Department of Radiation Oncology, University of Heidelberg, Im Neuenheimer Feld 400, Heidelberg 69120, Germany; 3 Department of Orthopedics, University of Heidelberg, Heidelberg, Germany; 4 Department of Hematology, Oncology and Rheumatology, University of Heidelberg, Heidelberg, Germany; 5 Interdisciplinary Tumor Center Mannheim, Mannheim University Medical Center, Mannheim, Germany; 6 Department of Radiology, University of Heidelberg, Heidelberg, Germany; 7 Department of Translational Oncology, National Center for Tumor Diseases, Heidelberg, Germany; 8 Institute of Pathology, University of Heidelberg, Heidelberg, Germany

**Keywords:** Soft tissue sarcoma, Extremity, Neoadjuvant chemotherapy, Intraoperative radiation therapy, Postoperative radiation therapy, Prospective trial

## Abstract

**Background:**

To report the results of a subgroup analysis of a prospective phase II trial focussing on radiation therapy and outcome in patients with extremity soft tissue sarcomas (STS).

**Methods:**

Between 2005 and 2010, 50 patients (pts) with high risk STS (size ≥ 5 cm, deep/extracompartimental location, grade II-III (FNCLCC)) were enrolled. The protocol comprised 4 cycles of neoadjuvant chemotherapy with EIA (etoposide, ifosfamide and doxorubicin), definitive surgery with IOERT, postoperative EBRT and 4 adjuvant cycles of EIA. 34 pts, who suffered from extremity tumors and received radiation therapy after limb-sparing surgery, formed the basis of this subgroup analysis.

**Results:**

Median follow-up from inclusion was 48 months in survivors. Margin status was R0 in 30 pts (88%) and R1 in 4 pts (12%). IOERT was performed as planned in 31 pts (91%) with a median dose of 15 Gy, a median electron energy of 6 MeV and a median cone size of 9 cm. All patients received postoperative EBRT with a median dose of 46 Gy after IOERT or 60 Gy without IOERT. Median time from surgery to EBRT and median EBRT duration was 36 days, respectively. One patient developed a local recurrence while 11 patients showed nodal or distant failures. The estimated 5-year rates of local control, distant control and overall survival were 97%, 66% and 79%, respectively. Postoperative wound complications were found in 7 pts (20%), resulting in delayed EBRT (>60 day interval) in 3 pts. Acute radiation toxicity mainly consisted of radiation dermatitis (grade II: 24%, no grade III reactions). 4 pts developed grade I/II radiation recall dermatitis during adjuvant chemotherapy, which resolved during the following cycles. Severe late toxicity was observed in 6 pts (18%). Long-term limb preservation was achieved in 32 pts (94%) with good functional outcome in 81%.

**Conclusion:**

Multimodal therapy including IOERT and postoperative EBRT resulted in excellent local control and good overall survival in patients with high risk STS of the extremities with acceptable acute and late radiation side effects. Limb preservation with good functional outcome was achieved in the majority of patients.

**Trial registration:**

ClinicalTrials.gov NCT01382030, EudraCT 2004-002501-72, 17.06.2011

## Background

Soft tissue sarcomas represent a rare tumor entity, accounting for less than 1% of all adult malignancies [1]. The cornerstone of curative intent treatment is surgery with negative margins. The addition of radiation therapy has been shown to distinctly improve local control, especially in patients with close/positive margins or high tumor grade [2], reaching 5-year local control rates of 80-90% after complete resection at least in extremity tumors [2]. Although long term local control can be achieved in the majority of patients, distant failure remains an unsolved issue occurring in about half of the patients, especially if risk factors like deep location, advanced tumor size and high tumor grade [3,4] are present, thus limiting 5-year overall survival to approximately 50-60% [3,5,6]. Therefore strategies with neoadjuvant and/or adjuvant chemotherapy have been investigated for high risk patients to eliminate occult metastases and assess chemosensitivity [7] by several investigators including our group. In 2004 we initiated a prospective one-armed clinical phase II trial on “Neoadjuvant therapy in patients with high risk soft tissue sarcoma” (NeoWTS trial, ClinicalTrials.gov NCT01382030, EudraCT 2004-002501-72) to investigate a multimodal approach consisting of neoadjuvant chemotherapy with etoposide, ifosfamide and adriamycin (EIA) followed by surgery, intraoperative electron radiation therapy (IOERT), postoperative external beam radiation therapy (EBRT) and adjuvant chemotherapy using the same regimen in patients with high risk soft tissue sarcoma. The main results of the trial regarding the primary endpoint (disease-free survival) and secondary endpoints (feasibility, response to neoadjuvant chemotherapy, time to progression, overall survival and chemotherapy associated toxicity) have been recently published by Schmitt et al. [7] and regarding prediction of chemosensitivity using fluorine-18-fluorodeoxyglucose positron emission tomography (FDG-18-PET) by Dimitrakopoulou-Strauss et al. [8]. Results regarding local control or side effects mainly attributable to local therapy (surgery, IOERT and postoperative EBRT) have not been addressed in detail in the prior publications. However, these parameters can strongly be influenced by tumor site. Whereas surgery and radiation therapy are frequently less challenging in extremity sarcomas, both treatment modalities are often compromised regarding the radicality of resection or the ability to achieve adequate target coverage during (postoperative) radiation therapy in non-extremity regions [9]. Consequently worse outcomes have been described for example in patients with retroperitoneal sarcomas [10], which showed significantly increased rates of margin positive resections and local failures compared to other sites. Although a similar distant metastasis rate was found, this resulted in an inversion of failure patterns in favor of local progression and a worse disease specific survival [10]. Local therapy associated side effects also depend strongly on the tumor region as they are mainly caused by directly adjacent organs at risk. For these reasons and to simplify comparisons with other published trials, which frequently report site-specific results, non-extremity tumors were excluded from the current analysis. Here we present the results of our prospective phase II trial focusing on local outcome and local therapy side effects in the subgroup of patients suffering from extremity tumors.

## Methods

Between 2005 and 2010 fifty-one patients with histologically proven potentially curable high risk soft-tissue sarcomas have been included into a prospective phase II trial on “Neoadjuvant Therapy in Patients with High-Risk Soft Tissue Sarcoma” (NeoWTS Trial, Clinical Trials.gov NCT01382030, EudraCT 2004-002501-72). Details regarding the study protocol, study design, statistical considerations, inclusion/exclusion criteria have been published already elsewhere [7,8]. In brief, high risk was defined as tumor size >5 cm, high grade (grade II/III according to the Federation Nationales des Centres de Lutte Contre le Cancer (FNCLCC)), deep or extracompartimental localisation, local relapse or inadequate previous therapy. Inadequate previous therapy was defined as an initial, non-oncological surgical procedure on the primary tumor. Tumors with size <5 cm after such procedures were also eligible, as per study protocol. Eligiliby criteria further included classical soft tissue sarcoma histology according to the world health organization (WHO) classification of soft tissue tumors, age 18–65 years, normal liver, renal cardiac and bone marrow function as well as Karnofsky-Index ≥ 80%. Histologies were centrally reviewed by a reference pathologist (GM) and graded according to the FNCLCC system. The same pathologist graded the operative specimens for tumor necrosis according to Salzer-Kuntschik [11]. The study was carried out according to Good Clinical practice (GCP) and the principles set in the Declaration of Helsinki 1964 as well as all subsequent revisions. The study protocol was approved by the corresponding institutional ethics committee (Independent ethics committee of the medical faculty at the University of Heidelberg) and legal authorities. All patients gave written informed consent to participate in the study.

### Population of current analysis

Thirty-five of the enrolled patients suffered from extremity soft tissue sarcomas. Extremity tumors were defined as tumors arising from the lower limb until the iliac crest or from the upper limb until the outer margin of the scapula. Patients with non-extremity.tumors or tumors involving the inner pelvic area or the thoracic space were excluded. One patient with extremity tumor was further excluded because she received an amputation after neoadjuvant chemotherapy and was therefore not scheduled for radiation therapy, leaving 34 evaluable patients for the current analysis.

### Imaging studies

Staging prior to therapy consisted of MRI and/or CT scans of the primary tumor region, FDG-18-PET and chest CT to exclude distant metastases. Tumor response was graded according to Response Evaluation Criteria in Solid Tumors (RECIST) by a radiologist experienced in musculoskeletal imaging. Follow up exams with MRI and/or CT scans were scheduled for every 2 cycles of chemotherapy, preoperatively, postoperatively, after study completion for every 3 months for the first two years, every six months for the following 3 years and annually thereafter.

### Planned treatment

The planned treatment consisted of 4 cycles of neoadjuvant chemotherapy using Etoposide, Ifosfamide and Adriamycin (EIA regimen), followed by definitive surgery with IOERT, postoperative EBRT and 4 cycles of adjuvant chemotherapy using the same regimen. Details regarding the chemotherapy regimen have been published already elsewhere [7]. In case of tumor progression after 2 cycles of neoadjuvant chemotherapy, the patient was referred to immediate local therapy with no further chemotherapy. Definitive surgery was planned four weeks after completion of neoadjuvant chemotherapy. IOERT and postoperative EBRT have not been further specified in the study protocol. The recommended dose was calculated for each patient under consideration of the individual situation and nearby structures at discretion of the treating radiation oncologist. Data regarding detailed radiation therapy parameters including radiation related toxicities and functional outcome were cross-checked and completed by review of patients’ charts and radiation therapy documentation.

### IOERT

IOERT was performed in a dedicated surgical theatre with an integrated Siemens Mevatron ME linear accelerator (Siemens, Concord, USA) capable of delivering 6–18 MeV electrons and thus covering a depth up to 6 cm. After the surgical procedure, an applicator of appropriate size was chosen to encompass the target area which was defined in correspondence with the treating surgeon. The applicator was manually positioned and attached to the table. Uninvolved radiosensitive tissues like major nerves and skin were displaced or covered by lead shielding whenever possible. The applicator was aligned with the linear accelerator using a laser guided air-docking system. The IOERT dose was prescribed to 90% isodose, which covered the whole surgical tumor bed with a safety margin of 1 cm. In case of a very large tumor bed, which could not be covered by a single applicator, either multiple fields were used or the intraoperative target volume was restricted to the area at highest risk for close or positive margins according to the treating surgeon. A dose of 15 Gy was attempted but could be reduced to 10–12 Gy at the discretion of the treating radiation oncologist if uninvolved radiosensitive structures at risk for severe radiation toxicity (e.g. major nerves) could not be removed from the irradiation field.

### External beam radiation therapy (EBRT)

External beam radiation therapy was performed by linear accelerators using CT-based 3D-conformal techniques in all patients. Patients were treated using multiple field techniques. At our institution, the target volume included the surgical volume with a safety margin of 2 cm in axial direction and 4 cm in longitudinal direction. Margins could be reduced in case of anatomical borders like uninvolved bones. The surgical scar was included into the irradiation field and at least one third of the circumference of the extremity was spared from irradiation to prevent chronic lymph edema whenever possible. A total dose of 40–50.4 Gy was attempted after IOERT depending on IOERT dose and resection margin at the discretion of the treating radiation oncologist. In patients, who did not receive an anticipated IOERT boost, postoperative radiation therapy included an external beam boost to the surgical bed with a margin of 1–2 cm in all directions to a total dose of ≥ 60 Gy. Conventional fractionation (single dose 1.8-2 Gy) was used in all cases.

### Definition of events and statistical considerations

Local control (LC) was defined as absence of tumor regrowth after surgery in the primary tumor region. Distant control (DC) was defined as absence of nodal or distant metastases. Disease-free survival (DFS) was defined as absence of local/distant failure and death from any cause. Overall survival (OS) was defined as absence of death from any cause. LC, DC and DFS were calculated from the date of definitive surgery until the corresponding event or the last follow-up information. OS was calculated from the first day of treatment until death or the last follow-up information. All time to event data was calculated using the Kaplan-Meier method. Toxicity was scored using the Common Terminology Criteria for Adverse Events (CTCAE) V3.0.

## Results

A total of 34 patients have been included into the current analysis. All patients received neoadjuvant chemotherapy, definitive surgery and radiation therapy. For detailed patient characteristics see Table 1. The median follow up for the entire cohort from inclusion into the trial was 43 months (9–80 months) and 38 months (6–78 months) from the date of surgery. Median follow up in survivors was 47 months from inclusion and 43 months from surgery.

**Table 1:** Patient characteristics

**Patient characteristics**	**N**	**%**
**Age**		
Median	52	
Min	37	
Max	65	
**Gender**		
Male	23	68
Female	11	32
**Localisation**		
Upper limb	4	12
Lower limb	30	88
**Histology**		
Liposarcoma	10	29
Synovial sarcoma	7	21
NOS	6	18
MFH	6	18
Leiomyosarcoma	1	3
Other	4	12
**Grading**		
FNCLCC grade 2	16	47
FNCLCC grade 3	18	53
**Size at FD**		
5-10 cm	17	50
> 10 cm	17	50
**Prior surgery**		
Biopsy only*	26	76
Previous surgery**	8	24

n: number of patients,%: percentage, age:[years], NOS: sarcoma not otherwise specified, MFH: malignant fibrous histocytoma, FNCLCC: Federation Nationales des Centres de Lutte Contre le Cancer, FD: first diagnosis, cm: centimeter, *: no surgery except incisional biopsy for pathological diagnosis, **: non-oncological surgical procedures.

### Response to neoadjuvant chemotherapy

Although at least minor tumor shrinkage was observed in the majority of patients, according to RECIST criteria most patients showed stable disease on imaging and poor response (defined as > 10% vital tumor) according to the pathological specimen. For detailed information about response see Table 2.

**Table 2:** Response to neoadjuvant treatment

**Reponse on imaging (RECIST)**	**N**	**%**
CR	2	6
PR	7	21
SD	22	65
PD	3	9
**Tumor necrosis (Salzer-Kuntschik)**	**N**	%
1 (no vital tumor)	5	15
2 (single vital tumor cells)	3	9
3 (vital tumor < 10% )	1	3
4 (vital tumor 10-50%)	9	26
5 (vital tumor > 50%)	15	44
6 (completely vital tumor)	1	3

n: number of patients,%: percentage, RECIST: response evaluation criteria in solid tumors, CR: complete remission, PR: partial remission, SD: stable disease, PD: progressive disease.

### Surgery

Definitive surgery was performed in all patients. Surgical procedures consisted of attempted wide excisions in 33 patients (97%), whereas one patient received a planned marginal excision to prevent a major functional deficit. Resection of the fibular nerve or its major branches was needed in 4 patients with lower extremity sarcoma. Two patients received an endoprothetic implant.

Negative margins (R0) were achieved in 30 patients (88%), while microscopic positive margins (R1) were found in 4 patients (12%). No patient had macroscopic residual disease. The minimal surgical margins after complete resection measured in the pathological specimen were <0.5 cm in 17 cases, 0.5-1 cm in 6 cases, and 1–2 cm in 2 cases. In 5 patients no vital residual tumor was found.

### IOERT

Intraoperative radiation therapy was performed as planned in 31 of the 34 patients (91%). Three patients did not receive IORT due to patient refusal or technical reasons. IOERT was performed with a median dose of 15 Gy, a median electron energy of 6 MeV and a median cone size of 9 cm. For detailed IOERT characteristics see Table 3. Major nerves had to be included in the IOERT volume in 12 patients. In 9 of these cases, the IOERT dose was therefore restricted to 10–12 Gy.

**Table 3:** Radiation therapy characteristics

**Radiation therapy**	**N**	**%**
**IOERT dose**		
10 Gy	3	10
12 Gy	8	26
15 Gy	20	65
**IOERT energy**		
6 MeV	21	68
8 MeV	8	26
10 MeV	2	6
**IOERT cone**		
circle-shaped	13	42
squizzle-shaped	18	58
median size	9 cm	
min size	5 cm	
max size*	22 cm	
**EBRT total dose**		
< 40 Gy	1	3
40-50.4 Gy	29	85
> 50.4**	4	12

n: number of patients,%: percentage (IOERT n = 31, EBRT n = 34), IOERT: Intraoperative electron radiation therapy, EBRT: external beam radiation therapy, Gy: Gray, MeV: mega electron volts, cm: centimeter, min: minimum, max: maximum, *: sum of multiple fields, **: one patient received two additional fractions for compensation of a planned treatment break for a total dose of 54 Gy, three patients without IORT boost received a total dose of 60 Gy.

### EBRT

All patients received EBRT postoperatively. The median time interval between surgery and start of EBRT was 36 days (range 22–158 days) and only 3 patients started EBRT more than 60 days after surgery. Reasons for delayed start of EBRT were wound complications in all of them, one therefore received postoperative CHT prior to postoperative radiation therapy. EBRT was performed using CT-based 3D-conformal treatment planning and conventional fractionation in all cases. Median EBRT dose was 46 Gy (range 20–54 Gy) in patients who had received an IOERT boost and 60 Gy in patients without. Median duration of EBRT was 36 days (range 13–50). EBRT was prematurely finished in one patient after 20 Gy according to patient’s choice. One patient had a planned treatment break >3 days during external beam radiation and received two additional fractions for compensation up to a total dose of 54 Gy. For detailed radiation therapy characteristics see Table 3.

### Local control

**Figure 1:**
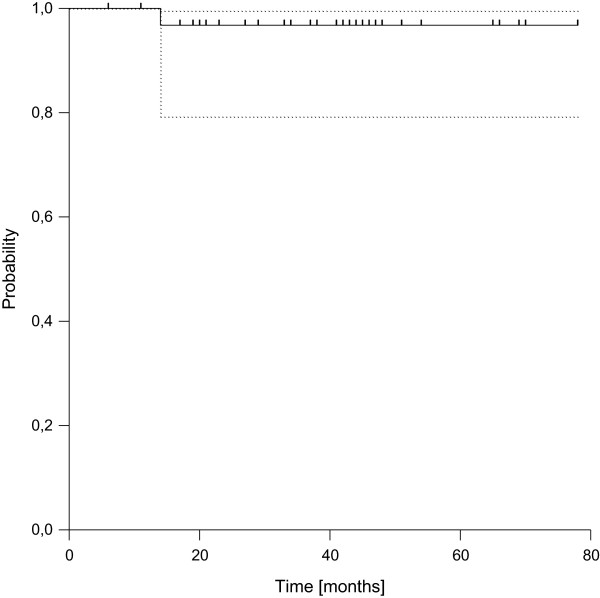
Local Control (dotted lines: 95% confidence interval).

Local recurrence was observed in one patient 14 months after definitive surgery. All other patients remained locally controlled, resulting in estimated 3- and 5-year local control rates of 97% (95%-confidence intervall: 79.2-99.5%, see Figure 1).

### Disease free survival and overall survival

**Figure 2:**
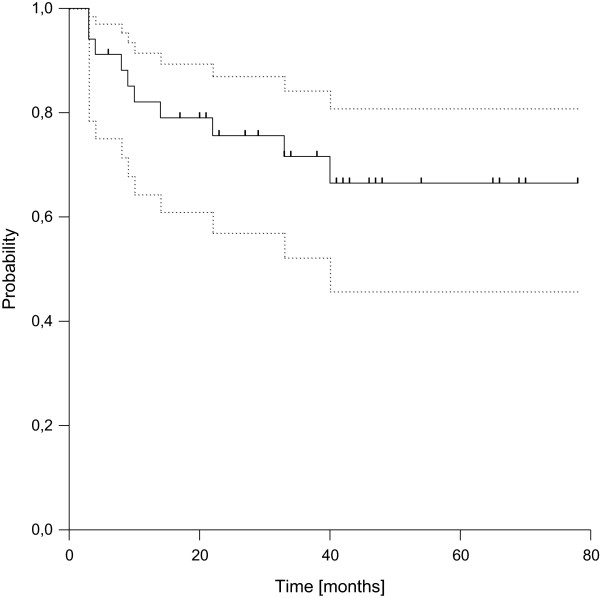
Disease-free Survival (dotted lines: 95% confidence interval).

**Figure 3:**
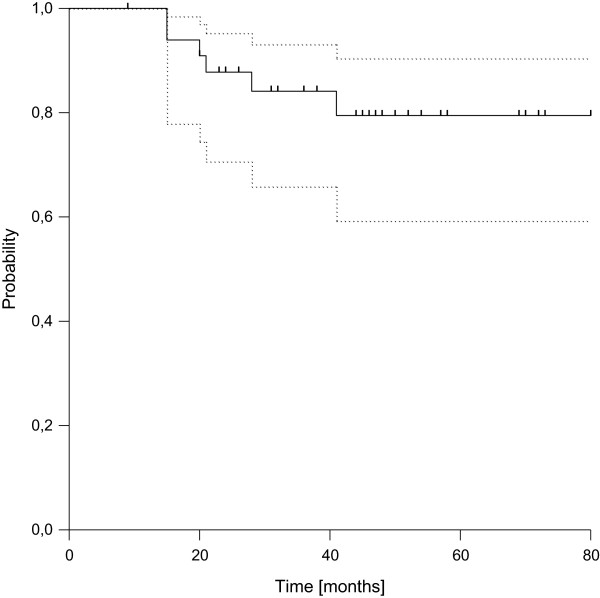
Overall Survival (dotted lines: 95% confidence interval).

Distant failure was found in 11 patients after 3 to 40 months (median 9 months). In 7 patients the initial site of failure was lung only (63%), whereas two patients developed lung and lymph node metastases at the same time and two patients suffered from nodal failure only. The resulting estimated 3- and 5-year disease-free survival rates were 72% (95%-CI: 52.2-84.2%) and 66% (95%-CI: 45.7-80.8%), respectively (see Figure 2). Three of the four patients with R1-resection failed distantly compared to 8 out of 30 in case of R0-resection, leading to a statistically significant difference in distant control and disease-free survival according to margin status (p = 0.017). Considering overall survival, we observed a total of 7 deaths, resulting in estimated 3- and 5-year rates of overall survival of 84% (95%-CI: 65.8%-93.1%) and 79% (95%-CI: 59.2%-90.4%), respectively (see Figure 3). All deaths were related to disease progression.

### Postoperative complications

Postoperative wound complications were observed in 7 patients (20%). These included two patients with non-infectious wound dehiscence (grade 1), two with seroma formation requiring puncture or drainage (grade 2), and three with abscess formation requiring intravenous antibiotics and/or re-operations (grade 3). Four patients had nerve resections and showed corresponding deficits postoperatively. Five additional patients had dys-/paresthesia outside the scar region postoperatively, which resolved in three and persisted in two patients.

### Chemotherapy associated toxicity

Overall, chemotherapy was well tolerated. Severe toxicity (defined as grade ≥ 3) was observed mainly as haematological side effects in 13 of 34 patients (38%), including three patients with grade 4 reactions. No severe renal, cardiac or hepatic toxicity were found. Cycle delays were needed in four and dose reductions in two patients. For detailed information see Table 4.

**Table 4:** Severe chemotherapy associated toxicity

**CHT toxicity**	**Grade 3**		**Grade 4**	
	**n**	**%**	**n**	**%**
Leukopenia	6	18%	3	9%
Anemia	6	18%		
Thrombopenia	1	3%		
Nausea/vomiting	2	6%		
Neurological	2	6%		
Neutropenic fever	2	6%		
Hemolysis	1	3%		
Diarrhea	1	3%		

CHT: chemotherapy, n: number of patients,%: percentage, toxicity of neoadjuvant and adjuvant cycles pooled together, some patients had more than one toxicity.

### Acute toxicity during EBRT

Mild radiation dermatitis (grade 1) was observed in the majority of patients (n = 18) during postoperative radiation therapy. Eight patients (24%) developed grade 2 radiation dermatitis, but none showed grade 3 dermatitis. Slight increases in lymph edema during adjuvant radiotherapy were observed in 6 patients and one patient developed a venous thrombosis.

Four patients receiving postoperative chemotherapy developed radiation recall dermatitis. Recall dermatitis developed 14–41 days from the last day of irradiation during the first or second cycle of adjuvant chemotherapy after complete restitution of acute radiation induced skin reaction. Two patients showed mild reactions (grade 1), while 2 patients had moderate dermatitis (grade 2). Radiation recall dermatitis resolved in all patients until the following chemotherapy cycle without dose reductions. None of the patients developed a recurrence of recall dermatitis during the following cycles. Onset of recall dermatitis was not correlated with the severity of skin reaction during EBRT (none had grade 2 skin reaction).

### Late toxicity

Mild to moderate late toxicities were observed in the majority of patients, mainly as hyperpigmentation of skin. Severe late toxicity was observed 6 patients (18%). For detailed information see Table 5. In particular, one patient suffered from new onset neuropathy with partial paresis, one from deep vein thrombosis and two patients from severe impairment of joint function. Two patients required surgical revisions due to late toxicity, one due to infection and dysfunction of a prosthetic implant and one after bone necrosis with fracture.

**Table 5:** Late toxicity

**Late toxicity**	**All grades**		**Grade 3**	
	**n**	**%**	**n**	**%**
Hyperpigmentation	20	59%		
Fibrosis	10	29%		
Neuropathy	4	12%	1	3%
Bone necrosis	2	6%	1	3%
Bursitis	2	6%		
Infection of implant	1	3%	1	3%
Deep vein thrombosis	1	3%	1	3%
Lymph edema				
At 1 year	10	33%*		
At 2 years	4	15%°		
Joint function				
At 1 year	11	37%*	2	7%*
At 2 years	6	23%°	1	4%°

n: number of patients,%: percentage, *: of 30 evaluable patients, °: of 26 evaluable patients, some patients had more than one toxicity.

### Functional outcome

Overall functional outcome was good in the majority of patients. At one and two years after surgery, 4 of 30 (13%) and 4 of 26 (15%) evaluable patients had severe impairment of limb function (defined as interfering with ADL), respectively. The cumulative incidence including patients with shorter follow-up or improvement of function over time was 6 of 34 (18%) in the first year and 8 of 34 (23%) in 2 years from surgery, including two secondary amputations.

Secondary amputations were needed in two patients (6%), both disease-related. One amputation was performed due to a local recurrence, which has been described above (see local control paragraph). The second patient was a 44 year old male with a 7 cm high grade sarcoma (histologically not otherwise specified, FNCLCC grade 3) at the lower lateral thigh. According to the protocol he was scheduled for 4 cycles of neoadjuvant chemotherapy but showed progressive disease after 2 cycles and went on to local therapy directly. Wide excision with free margins was achieved including IOERT with 15 Gy using a 9 cm cone, followed by EBRT with 45 Gy. 8 months after the end of EBRT, suspicious lymph nodes were discovered during routine follow up in the inguinal and iliacal region. They were surgically removed and found positive for disease while an incisional biopsy of the primary tumor region revealed no local recurrence. Later on, the patient developed a massive recurrence in the nodal areas complicated by a large haematoma and was treated with hemipelvectomy. The final pathology assessment of the hemipelvectomy specimen confined the lymph node recurrence but revealed no vital tumor in the primary tumor region.

In summary, long term limb preservation was achieved in 32 patients (94%) with good functional outcome (no interference with activities of daily life) in 81% of them.

## Discussion

Here we report the results of a subgroup analysis of a prospective, single institution, non-randomised trial which investigated a complex multimodal treatment approach consisting of neoadjuvant chemotherapy followed by surgery, intraoperative electron radiation therapy, postoperative external beam radiotherapy and postoperative chemotherapy in high grade soft tissue sarcoma limited to patients suffering from extremity tumors.

Since Rosenberg et al. [12] described a similar overall survival comparing amputation with limb sparing surgery followed by radiation, the combination approach has emerged as the standard of care in extremity sarcomas with high risk features. Although radiation therapy results undoubtedly in increased rates of local control [2], high doses of ≥ 60 Gy need to be prescribed to large volumes in many patients which can be associated with marked acute and late toxicities and consequently result in unfavourable functional outcomes. Intraoperative radiation therapy is a treatment technique, which has been developed for dose escalation in body regions, where such doses are hardly achievable with external beam radiotherapy alone because of adjacent organs at risk which much lower tolerance than in extremity regions. However intraoperative radiation therapy has been introduced by several groups including ours also in the treatment of extremity tumors [13-16] to replace the external beam boost mainly because of its unique opportunity to guide a high single dose directly to the high risk region for close or positive margins under visual control during surgery. Further advantages in comparison to an external boost include at least theoretically smaller field sizes (because safety margins for daily positioning errors can be omitted), the possibility to exclude organ at risk like major nerves or skin from the irradiation field and the reduction of overall treatment time. Therefore a combination of limb sparing surgery, IOERT and EBRT according to our institutional standard was included as local therapy component also into our prospective phase II trial. With a completion rate of 91% of the planned IOERT procedures and 97% of the planned EBRT procedures we could show that this combination can be integrated easily into a prospective trial even using a complex multimodal treatment regimen at least in an experienced tertiary reference center. Although not further specified in the protocol, the applied doses during IOERT and EBRT were relatively homogenous based on our standard operating procedures for clinical routine use. The same was true regarding compliance to the EBRT component. Start of EBRT had to be postponed only in 3 of 34 patients due to postoperative wound complications in all of them and no unplanned treatment breaks > 3 days were necessary.

Using this approach, we observed an excellent 5-year local control rate of 97% and encouraging 5-year rates of disease-free (66%) and overall survival (79%) with acceptable acute and limited late toxicity transferring into high rates of long-term limb preservation (94%) with good functional outcome in the majority of patients (81%). These results seem to compare favourably with major retrospective series using similar combinations of intraoperative and external beam radiation therapy (see Table 6, [13-18], which reported consistently 5-year local control rates of 80-90%, although keeping in mind that the percentage of incomplete resections in our trial was lower than in most of these series (12% vs. 39-58%). This might be at least partly attributable to the use of neoadjuvant chemotherapy, although major responses according to RECIST criteria were rare. Further on, local control seemed to be at least slightly improved compared to recent series using EBRT alone (pre- or postoperatively), which reported consistently 5-year-LC rates of 83-93% [6,19-26] with mainly comparable R1-resection rates (0-25%) as in our trial. This might be attributable to the increased biological effect of the high single dose which was guided directly to the high risk region under visual control via IOERT, but given the limited number of patients in our study and the lack of a control arm, it cannot be ruled out that this difference has occurred by selection bias or randomly.

**Table 6:** Results of major IORT series

**Author**	**Year**	**n**	**f/u**	**R0 (%)**	**IORT**	**EBRT**	**5-y-LC**	**5-y-OS**	**LP (%)**	**FC (%)**
Edmonson [13]	2001	39	70	62	10-20	45	90*	80	95	n.r.
Azinovic [14]	2003	45	93	67	15	45-50	80*	64*	88	77
Kretzler [15]	2004	28	52	61	12-15	50	84	66	100	59
Llacer [17]	2006	79	58	42	20 (LDR)	45-50	90	69	100	n.r.
Oertel [16]	2006	128°	33	49	15	45	83	83	90	86
Alvarez [18]	2008	53	66	n.r.	7.5-12.5	n.r.	87	75	83	81
Current Study	2013	34	43	88	10-15	40-50	97	79	94	81

n: number of patients, f/u: median follow up,%: percentage, R0: rate of microscopic complete resections, IORT: intraoperative radiation therapy dose in Gy, EBRT: external beam radiation therapy dose in Gy, 5-y-LC: estimated 5-year-local control rate in%, 5-y-OS: estimated 5-year-overall survival rate in%, LP: limb preservation rate, FC (%): rate of excellent/good functional outcome, *: crude rates, °: excluding patients with distant metastases at time of surgery.

Aside from local control, there is an ongoing debate not only about the value of additional boosting techniques like IOERT or brachytherapy, but also about the timing of EBRT, which is driven mainly by functional issues. In the initial report on the prospective randomized trial comparing preoperative and postoperative EBRT conducted by the NCI Canada, increased rates of wound complications but reduced rates of acute skin toxicity were found in the preoperative arm [27]. Subsequent analyses with longer follow up failed to show significant differences in oncological endpoints, but reported significantly lower rates of severe fibrosis and trends for reductions of severe edema and joint stiffness [28,29] with preoperative radiation therapy. Although functional outcome analysis revealed no significant differences between the treatment arms, severe fibrosis, edema and joint stiffness were associated with lower functionality scores in general and their onset increased with field size [29]. Stinson et al. [30] also reported associations between increased total dose and/or field size with late toxicities like pain, edema, decreased muscle strength or range of motion in postoperatively irradiated patients.

Compared to postoperative EBRT alone, introduction of an IOERT boost instead of the percutaneous boost phase should lead also to a reduction in field size at least regarding the high dose areas, which may consequently result in reduced late toxicity and improved functional outcome. In contrast to preoperative EBRT, a markedly increased wound complication rate compared to postoperative EBRT alone seems unlikely, because the skin is excluded from the boost area. These assumptions were, at least in part, supported by our results.

We observed a wound complication rate of 20% in our study, which is similar to series using postoperative EBRT alone [27,31] and compares favourably with series using preoperative EBRT [19,27] indicating that the use of an IOERT boost does not increase the wound complication rate. Further on, the rate of acute radiation related side effects was similar to the preoperative arm of the NCI trial and compares favourably with series using postoperative EBRT alone [27,31], which is probably related to the reduced EBRT doses by omitting the external boost phase.

Interestingly, we observed 4 cases (11%) of radiation recall dermatitis during the adjuvant chemotherapy phase. Radiation recall dermatitis is a poorly understood acute inflammatory skin reaction confined to previously irradiated areas, which occurs triggered by drugs, especially chemotherapy agents, after prior complete restitution of acute radiation related side effects. Although its appearance has been described in association with many commonly used chemotherapy substances [32], only few systematic reports have examined its incidence. Kodym et al. [33] reported an observational study of 91 patients who received different chemotherapy regimens after radiation therapy for bone metastases of which 8 (9%) developed recall dermatitis, but did not find an association with a particular substance or substance group. However, based on the rare available data, adriamycin seems to be one of the substances with an increased risk for recall dermatitis [32]. For example, Haffty et al. [34] described recall dermatitis in 15 of 148 patients (10%) who received mainly adriamycin based chemotherapy after accelerated partial breast irradiation. Because to our knowledge, no cases of recall dermatitis have been described in the literature triggered by ifosfamide and only one for etoposide [32], it seems likely that the cases in our study were elicited by adriamycin. Several authors have recommended withdrawal, delay or dose reductions of the triggering agent although there is limited evidence supporting these strategies, because even re-challenge with the same drug does not necessarily elicit a recurrent reaction [35]. Because in our study none of the reactions were severe, all patients were re-challenged without dose reductions during the following cycles and none developed a recurrence of recall dermatitis.

The overall rate of severe late toxicity found in our study was in the range of other series (3% to >22%) reporting on patients treated with surgery and radiation for extremity sarcomas without IOERT [23,30] and similar to the findings of a previous large retrospective analysis of our group with IOERT [16]. Nevertheless we observed considerable rates of fibrosis, joint stiffness and lymph edema, although similar or even higher rates have been reported by others using postoperative EBRT alone. For example, Davis et al. [29] described fibrosis ≥ grade 2 in 48%, joint stiffness in 23% and edema in 23% of the patients treated with postoperative EBRT and Alektiar et al. [31] found 39% joint stiffness and 32% edema even using intensity-modulated radiation therapy. However, we also found a decrease in overall rate and severity of lymph edema and joint stiffness over time, probably related to ongoing physical therapy as described by others [31], which further complicates any comparison. In this context it should be mentioned, that IOERT volume itself was shown as the only factor significantly associated with severe fibrosis in the study of van Kampen et al. [36] and therefore should be restricted to the justifiable minimum.

The same seems true for fractures and neuropathy, which have been described as dose limiting side effects for IOERT in other parts of the body [37]. In our study, one patient (3%) developed a fracture, which is in the range of reported rate (1-8%) with [14,38] or without IOERT [31,39,40] as part of radiation therapy. However, as fractures may occur many years after the end of radiation treatment as highlighted by a large analysis from Gortzak et al. [40], it cannot be ruled out that the fracture rate is underestimated by our analysis due to the relatively short follow up. Considering radiation related neuropathy, we observed 4 cases in total (12%) with severe grade in one (3%) in the study population. Again, similar rates have been reported in reports using EBRT alone [41]. Alektiar et al. [31] even observed a rate of 28% in total of which 5% were grade 2/3 with postoperative IMRT. However, if only the 12 patients with inclusion of major nerves into the IOERT field were analysed, the neuropathy rate increased to 25% (8% severe) in our study, which is similar to the findings of Azinovic et al. [14] using also a combination of IOERT and EBRT, thus indicating that major nerves should be excluded from IOERT fields whenever possible.

The role of chemotherapy in the treatment of high-risk sarcomas with curative intent remains controversial as several randomized trials and meta-analyses have reported conflicting results. In the adjuvant setting, two major phase III trials conducted by the EORTC (62771 and 62931) [42,43] have failed to show a significant benefit for overall survival with the addition of chemotherapy. While the first one reported at least a significant benefit in relapse-free survival, this result could not be confirmed in the latter one. Further on, the observed improvement in relapse-free survival in EORTC 62771 was based mainly on fewer local relapses in the CHT group without a significant difference in the frequency of distant metastasis [42]. As a consequence, local therapy was intensified in the second trial and significant differences in local and overall relapse free survival were no longer observed [43]. Thus one may, argue that the value of adjuvant chemotherapy could be mainly based on counterbalancing an inadequate local therapy while marked improvements seem unlikely in patients with appropriate local treatment. In contrast, two randomized trials from Italy reported significant improvements in overall survival for the addition of adjuvant chemotherapy [44,45]. Although comparisons between different trials are always difficult, interestingly the 5-years overall survival rates of the CHT arms were similar between the EORTC (63% and 67%) and the Italian trials (66% and 70%), while the control groups showed marked differences (56% and 67% in the EORTC, 46% and 47% in the Italian trials), indicating that the different outcome of the control arms might have influenced the conflicting results. However, the initial SMAC meta-analysis [46] also reported a significant benefit for the use of perioperative chemotherapy in terms of local/distant failure free interval and relapse free survival although it failed to show a significant difference in overall survival. Interestingly, patients with extremity tumors (which usually allow higher rates of intensified local treatments compared to other body regions) showed the largest benefit from additional chemotherapy, indicating that chemotherapy effects seem not restricted patients with inadequate local therapy. An updated meta-analysis [47] adding several trials using more potent chemotherapy combinations confirmed the initial findings for relapse-free survival and showed also an improvement in overall survival, but did not include the recent negative EORTC trial.

Neoadjuvant approaches of chemotherapy with or without radiation therapy theoretically have several benefits including improved resectability with better functional outcome, histological response evaluation for further treatment stratification and early treatment of potentially occurred microscopic distant spread. Several non-randomized trials showed high rates of histological response [48,49] up to ~ 50%, which correlated with outcome including overall survival [48]. Delaney et al. [50] and Mullen et al. [51] also described excellent results in a highly unfavourable patient group after treatment with an intensified regimen of neoadjuvant chemoradiation at Massachusetts General Hospital (MGH), although considerable rates of toxicity were observed. Further on, this approach resulted in significant improvements in terms of local control, freedom from distant metastases, disease-free and overall survival compared to a historical control group treated without chemotherapy, highly indicating that a neoadjuvant approach might be beneficial. However, the only randomized trial comparing additional neoadjuvant chemotherapy with local therapy alone published by Gortzak et al. [52] in 2001 did not find any significant difference between the chemotherapy and the control arm. Further on, when the MGH approach was tested in a multi-institutional setting (RTOG 9514), toxicity was even higher and outcomes were clearly worse than expected from the MGH experience [22], although the results continued to compare well with historical data given the highly unfavourable group of patients included.

In our study using preoperative chemotherapy alone with radiation applied intra- and postoperatively, we observed a moderate clinical response rate, which was in the range reported by other groups using preoperative chemotherapy, chemo-hyperthermia or chemoradiation (11-29%) [22,50,53]. However, the pathological response rate (defined as <10% vital cells in our study) was lower than in many other series [22,48,50]. This might be due to the omission of preoperatively applied radiation therapy or the different chemotherapy schedule in our study. Nevertheless, our results compare well with many other series regarding local control and are in the range of other trials using more intensive neoadjuvant approaches with higher rates of pathological response in terms of disease-free and overall survival. Thus, local dose escalation via IOERT seems to be able to compensate for an unfavourable response to neoadjuvant chemotherapy at least regarding local control and a low rate of pathological treatment response might not necessarily result in a poor overall outcome.

Clearly, our study has some limitations, mainly due to the small patient number, the relatively short follow-up and the lack of a control arm. Further on, the study was initially designed mainly to evaluate short term effects of neoadjuvant chemotherapy and therefore did not include highly standardized specifications for local therapy or assessment of late side effects. Therefore conclusions should be drawn with caution. Nevertheless it represents prospectively collected data on the use of intraoperative radiation therapy embedded into a multimodal treatment approach, adding valuable information to the mainly retrospective evidence regarding this particular radiation technique.

## Conclusion

In summary, our approach consisting of neoadjuvant chemotherapy, limb sparing surgery, intraoperative and postoperative radiation therapy and adjuvant chemotherapy resulted in excellent local control rates and good disease-free and overall survival in patients with high risk extremity sarcomas, although objective pathological response rates to neoadjuvant chemotherapy were only moderate. Inclusion of an intraoperative radiation boost into this complex multimodal approach seemed easily manageable with high rates of local treatment compliance. With this approach we observed low rates of acute and acceptable rates of late toxicities transferring into a high limb preservation rate with good functional outcome. However, given the limitations of our study, the real extent of possible benefits using additional boosting techniques like intraoperative radiation therapy compared to external beam radiation alone or neoadjuvant chemotherapy/chemoradiation compared to upfront surgery can only be further clarified in randomized trials.

## Competing interests

The authors declare that they have no competing interests.

## Authors’ contributions

FR drafted the manuscript, supervised intraoperative radiation treatment and participated in data acquisition, statistical analysis and literature review. BL participated in data acquisition, manuscript draft and supervised surgical treatment. TS participated in data acquisition, statistical analysis, literature review, medical treatment and drafting of the manuscript. BK participated in protocol design, data acquisition and medical treatment. GE supervised protocol design and medical treatment. OS supervised response evaluation on imaging according to RECIST. CG participated in data acquisition and medical treatment. GM served as a reference pathologist and graded postoperative tumor specimen according to Salzer-Kuntschik. PW participated in data acquisition and medical treatment. FWH supervised intraoperative radiation therapy physics. PEH and JD revised the manuscript critically. MB participated in data acquisition, statistical analysis and literature review, supervised external beam radiation therapy and revised the manuscript critically. All authors read and approved the final manuscript.

## Authors’ information

Falk Roeder and Burkhard Lehner, shared first authorship.

## Pre-publication history

The pre-publication history for this paper can be accessed here:

http://www.biomedcentral.com/1471-2407/14/350/prepub
